# From lionizing to protecting health care workers during and after COVID‐19—systems solutions for human tragedies

**DOI:** 10.1002/hpm.3138

**Published:** 2021-03-01

**Authors:** Ethan Guillén, Marine Buissonnière, Christopher T. Lee

**Affiliations:** ^1^ Prevent Epidemics Resolve to Save Lives, an Initiative of Vital Strategies New York USA

**Keywords:** COVID‐19, health care workers, health policy health security, infection prevention and control, WASH

## Abstract

During the COVID‐19 pandemic, health care workers (HCWs) have been lauded as heroes, yet both before and during the pandemic, they lacked the protections needed to keep them safe. We summarize data on HCW infections and deaths during previous epidemics, the costs of the failure to protect them, and provide recommendations for strengthening HCW protections by investments in and implementation of infection prevention and control and water, sanitation, and hygiene programs, training and career development, and national and global monitoring of HCW infections. We must move from placing individuals at undue risk to accepting collective responsibility and accountability for the well‐being of our HCWs and take concrete actions to protect HCWs who risk their lives to protect patients and populations.

## 1 INTRODUCTION

Even as the world lionizes health care workers (HCWs) as heroes, we fail to keep them safe. The COVID‐19 pandemic made this contradiction more apparent than ever. As the virus swept the globe in early 2020, public displays of support for overburdened HCWs were common. Yet, the lack of protections for HCWs—and their consequences—were clear: hundreds of thousands of HCW infections, reports of HCWs wearing trash bags in the absence of proper personal protective equipment (PPE), and an uptick in mental health issues and suicides as HCWs shouldered the strain of ill‐prepared public health systems.

Failures to protect HCWs are not new or unique to the COVID‐19 pandemic. HCW infections threaten control of outbreaks by sidelining necessary staff; making HCWs (and, by proxy, health facilities) vectors for spreading disease; depleting the health care workforce; and impeding the regular provision of routine care. We summarize data on risks to the health care workforce from several major epidemic events over the last 2 decades, the costs of failing to protect HCWs, and propose key recommendations for national and global action.

## 2 THE HEALTH CARE WORKFORCE IS AT RISK

In 2014, the World Health Organization (WHO) reported a global shortage of 7.2 million HCWs, with 83 countries characterized as having a staffing crisis, and that an additional 18 million HCWs would be needed in the next decade.^1^ The rapid increase in infectious disease outbreaks in recent years represents a growing threat to the already strained health care workforce.^2^ These impacts extend to the general population, who are often unable to access needed health services due to illness and death among HCWs.^3^ Failing to protect HCWs weakens health care systems and hinders achieving the goal of universal health coverage.

Due to a lack of consistent reporting, the total number of COVID‐19 infections and deaths among HCWs is unknown. As of August 2020, the International Council of Nurses reported 572,478 HCWs from 32 countries were infected with SARS‐CoV‐2, comprising approximately 10% of confirmed infections.^4^ Similarly, WHO found that in Europe and the Americas, 14% of reported COVID‐19 cases were among HCWs, despite comprising just under 3% of the population of lower‐ and middle‐income countries and 8% in higher‐income countries.^5^ Extrapolating from these data, the International Council of Nurses estimated at least two million HCW infections worldwide. In February 2021, WHO estimated that at least 30,000 HCW had died from COVID‐19.^6^

Although COVID‐19 has drawn global attention to the issue of HCW infections, multiple epidemics over the past 2 decades also put HCWs at risk (Table 1). In addition to becoming ill themselves, HCWs played a significant role in transmitting the virus in hospitals: 55% of likely SARS cases in Taiwan and 72% in Toronto were linked to hospital infection.^21^

**TABLE 1:** Infections among health care workers (HCW) during epidemics, 2002–2020

Years	Pathogen	HCW infections	Proportion of all infections	HCW deaths	Increased likelihood of HCW infection
2019‐	SARS‐CoV‐2	572,478 in 32 countries^4^	Unknown	At least 7000^6^	3.1^7^ ^,^ ^a^ ^,^ ^8^ ^,^ ^b^
2018–2020	EVD (DRC)^9^	171	5%	At least 41^10^ ^,^ ^c^	Unknown
2018–2020^d^	Lassa (Nigeria)^11^ ^,^ ^12^ ^,^ ^13^ ^,^ ^14^	109	4%	Unknown	Unknown
2014–2016	EVD (Guinea, Liberia, Sierra Leone)	881^15^	3%	513^15^	21‐32^16^
2012‐Present	MERS^17^, ^18^	415^17^ ^,^ ^e^ ^,^ ^19^	16%^‡^	25	Unknown
2009–2010	Influenza A (H1N1) pandemic^20^	Unknown	Unknown	Unknown	1.93‐2.52^f^
2002–2003	SARS^19^	1706	21.07%	Unknown	Unknown

Abbtreviations: DRC, Democratic Republic of the Congo; EVD, Ebola virus disease.

^a^ In Wuhan, China.

^b^ Similar results were found in Britain where a multivariate‐adjusted hazard ratio of 3.4 was calculated.

^c^ As of 14 July 2020.

^d^ As of 27 Sept 2020.

^e^ As of 2 June 2018.

^f^ The study gives slightly different figures based on the comparison group.

### 2.1 The costs of the failure to protect HCWs

COVID‐19 has highlighted the primary and secondary effects of infections among HCWs. A forthcoming World Bank study seeks to provide empirical estimates of the costs resulting from higher levels of HCW absenteeism, loss of the HCW workforce, and increased healthcare‐associated infections resulting from HCW infections, as well as downstream loss of trust in health facilities, leading to disruptions to essential services. During the 2014–2016 West African Ebola virus disease epidemic in Sierra Leone, 7% of the health care workforce died, leading to a 23% reduction in health services.^15^ One study estimated that an additional 4022 women would die each year during childbirth as a result of HCW deaths.^3^

The International Monetary Fund estimated that US$11 trillion was spent as of June 2020 for COVID‐19 response, including new spending, lost revenues and loans to boost economies.^22^ McKinsey & Company estimated that preparations to prevent and fight future pandemics would cost US$70‐120 billion to establish over two years and US$20‐40 billion a year to maintain thereafter—not including costs of implementing infection prevention and control (IPC) and water, sanitation and hygiene (WASH) programs.^23^ As of December 2020, WHO reported that 1 in 4 health care facilities worldwide had no water services and, in the world's 47 least‐developed countries, 1 in 2 health care facilities had no basic drinking water,^24^ despite costing roughly US$1 per capita to establish basic water services in all health facilities.

### 2.2 Make facilities safer to make HCWs safer

Full implementation of IPC and WASH standards in all health facilities can occur by implementing WHO's 2019 ‘Minimum requirements for IPC programmes’ to protect HCWs and make health facilities safer. IPC consists of a hierarchy of measures to protect HCWs and the patients they serve. Administrative controls include universal screening and triage of all patients and standardized training of HCWs; environmental controls reduce the risk of exposure and include use of outdoor screening and triage areas to improve ventilation, as well as adequate distance between HCWs and patients; while PPE provides additional protections and is most effective in conjunction with other components of the hierarchy.

Both IPC and WASH are essential for day‐to‐day care, for preventing transmission of antibiotic‐resistant bacteria, and for preventing outbreaks small and large.^25^ Taken together, rigorous implementation of IPC and WASH prevents transmission of infections in health care settings, increases patient safety, and keeps HCWs from becoming patients themselves. Progress must be closely tracked to ensure that policy implementation moves from national to local facility levels.

### 2.3 Invest in training, tools and resources for a safer health care workforce

HCWs must receive training and tools to recognize disease syndromes to provide clinical care, report outbreaks, and implement appropriate standard and transmission‐based precautions required to prevent infection. HCWs must have access to, and sufficient knowledge to effectively and safely use, adequate PPE, which is critical at all times to protect HCW from daily risks from meningitis, tuberculosis, measles and other common pathogens. A recent economic analysis of PPE investment costs found that US$9.6 billion would adequately protect all HCW in low‐ and middle‐income countries, saving more than 2 million lives in those countries with a societal return on investment of US$755.3 billion, an 80‐fold return.^26^ To ensure an adequately trained workforce, countries should develop career paths for IPC, including certification programs through continuing medical education, similar to field epidemiology training programs that equip disease detectives to identify and stop outbreaks.

### 2.4 Improve and use tools to monitor implementation and enact further improvements at facility, national and global levels

Transparent evaluation systems enable benchmarking and monitoring of progress in implementing lifesaving practices that make health systems stronger while protecting HCWs. While strong tools exist, they must be improved to better evaluate progress in meeting IPC standards. Governments should implement monitoring frameworks at the national (using WHO's Infection Prevention and Control Assessment Tool) and facility levels, with supportive supervision for continuous quality improvement.

At the global level, WHO, working with governments, should review existing indicators in the Joint External Evaluation and State Party Self‐Assessment Annual Reporting tool to ensure they adequately account for IPC and WASH. Specifically, a new technical area for safe delivery of health care services should more effectively measure the ability to deliver adequate clinical care, as well as implementation of IPC and WASH in health facilities.^27^

Understanding of the risks faced by HCWs is hampered by a lack of data on HCW infections and deaths during outbreaks, which should be collected by governments as part of a core set of indicators on human resources for health, with annual reporting to WHO and published by the Global Health Observatory.

## 3 CONCLUSION

COVID‐19 has exposed a great paradox of health care—who protects the people who protect us? While every death is a tragedy, HCWs are at significantly higher risk of infection than the general population, and illness and death among HCWs impede delivery of essential health services. The costs of solving this problem must be borne not by individuals, but by the societies that they serve.

Recommendations directed towards both governments and WHO to protect HCWs—particularly those in the least developed countries—must be a priority for development partners and global financiers. Epidemic and pandemic outbreaks are not simply a matter of national responsibility, but rather, global solidarity, as many outbreak pathogens can quickly cross borders. As a simple matter of economics, the loss of trillions of dollars in the global economy can be prevented by a relatively small investment in programs to stop pandemics and keep HCWs safe. Private, governmental, and multilateral donors must increase and coordinate investments in IPC training, supplies and monitoring, including adequate WASH facilities, to support efforts to improve HCW safety as part of all program specific or overarching health systems investments.

The world's lack of preparedness for outbreaks of all sizes and refusal to adequately protect HCWs is a failure not only of governments, but also of international institutions and donors. We know what must be done. We must move from placing individuals at undue risk to accepting collective responsibility and accountability for the well‐being of our HCWs, and take concrete action to protect HCWs who risk their own lives to care for us all.

## ETHICS STATEMENT

The data in this manuscript are from previously published sources; this work did not constitute human subjects research

## Data Availability

All data are cited and publicly available.

## References

[1] A Universal Truth: No Health without a Workforce. Forum Report, Third Global Forum on Human Resources for Health, Recife, Brazil. Global Health Workforce Alliance and World Health Organization, 2013. https://www.who.int/workforcealliance/knowledge/resources/hrhreport2013/en. Accessed 28 Jan 2021.

[2] Smith KF , Goldberg M , Rosenthal S , et al. Global rise in human infectious disease outbreaks. J R Soc Interface. 2014 Dec 6;11(101):20140950. 10.1098/rsif.2014.0950. 25401184 PMC4223919

[3] Evans DK , Goldstein M , Popova A . Health‐care worker mortality and the legacy of the Ebola epidemic. Lancet Glob Health. 2015;3(8):e439‐e440. 10.1016/S2214-109X(15)00065-0. 26163833

[4] Protecting Nurses from COVID‐19 a Top Priority: A Survey of ICN's National Nursing Associations. International Council of Nurses; 14 Sept 2020. https://www.icn.ch/system/files/documents/2020‐09/Analysis_COVID‐19%20survey%20feedback_14.09.2020%20EMBARGOED%20VERSION_0.pdf. Accessed 28 Jan 2021.

[5] Coronavirus Disease (COVID‐19) . Data as Received by WHO from National Authorities, as of 11 October 2020, 10am CEST. World Health Organization; 12 Oct 2020. https://www.who.int/docs/default‐source/coronaviruse/situation‐reports/20201012‐weekly‐epi‐update‐9.pdf. Accessed 28 Jan 2021.

[6] Ghebreyesus TA . Vaccine Nationalism Harms Everyone and Protects No One. Foreign Policy; 2 Feb 2021. https://foreignpolicy.com/2021/02/02/vaccine‐nationalism‐harms‐everyone‐and‐protects‐no‐one/. Accessed 5 Feb 2021.

[7] Pan A , Liu L , Wang C , et al. Association of public health interventions with the epidemiology of the COVID‐19 outbreak in Wuhan, China. J Am Med Assoc. 2020 May 19;323(19):1‐9. 10.1001/jama.2020.6130 PMC7149375 32275295

[8] Nguyen LH , Drew DA , Graham MS , et al. Risk of COVID‐19 among frontline healthcare workers and the general community: a prospective cohort study. Lancet Public Health. 2020 Sep;5(9):e475‐e483. 10.1016/S2468-2667(20)30164-X. 32745512 PMC7491202

[9] Ebola Virus Disease Democratic Republic of Congo: External Situation Report 98/2020. World Health Organization; 24 June 2020. https://www.who.int/publications/i/item/10665‐332654. Accessed 28 Jan 2021.

[10] Soucheray S . Three More Health Workers Infected in Ebola Outbreak. Center for Infectious Disease Research and Policy; 11 July 2019. https://www.cidrap.umn.edu/news‐perspective/2019/07/three‐more‐health‐workers‐infected‐ebola‐outbreak#:∼:text=The%20new%20cases%20raise%20the,outbreak%2C%20according%20to%20DRC%20data. Accessed 28 Jan 2021.

[11] Dan‐Nwafor CC , Furuse Y , Ilori EA , et al. Measures to control protracted large Lassa fever outbreak in Nigeria, 1 January to 28 April 2019. Euro Surveill. 2019 May;24(20):1900272. 10.2807/1560-7917.ES.2019.24.20.1900272. PMC6530254 31115314

[12] 2019 Lassa Fever Outbreak Situation Report. Nigeria Centre for Disease Control; 29 Dec 2019. https://ncdc.gov.ng/themes/common/files/sitreps/8c02d1bf6e3e02aa2adfe144dda40db2.pdf. Accessed 28 Jan 2021.

[13] 2018 Lassa Fever Outbreak Situation Report. Nigeria Centre for Disease Control. 31 Dec 2018. https://ncdc.gov.ng/themes/common/files/sitreps/733d856bae4b2afa5d29c0465e6c335e.pdf. Accessed 28 Jan 2021.

[14] Lassa Fever Situation Report: Epi Week 39: 21‐27 September 2020. Nigeria Centre for Disease Control; 27 Sept 2020. https://ncdc.gov.ng/themes/common/files/sitreps/46ae3c66dda6ddf9248133d1c6aec38d.pdf. Accessed 28 Jan 2021.

[15] Cost of the Ebola Epidemic. Centers for Disease Control and Prevention. 8 March 2019. https://www.cdc.gov/vhf/ebola/history/2014‐2016‐outbreak/cost‐of‐ebola.html. Accessed 28 Jan 2021.

[16] Andrada C . Ebola Vaccine and the Crucial Role of Healthcare Workers. Outbreak Observatory; 9 Aug 2018. https://www.outbreakobservatory.org/outbreakthursday‐1/8/9/2018/ebola‐vaccine‐and‐the‐crucial‐role‐of‐healthcare‐workers#:∼:text=The%202014%2D%202016%20West%20African,affect%20the%20outcomes%20of%20patients. Accessed 28 Jan 2021.

[17] Elkholy AA , Grant R , Assiri A , Elhakim M , Malik MR , Van Kerkhove MD . MERS‐CoV infection among healthcare workers and risk factors for death: retrospective analysis of all laboratory‐confirmed cases reported to WHO from 2012 to 2 June 2018. J. Infect. Public Health. 2020 Mar;13(3):418‐422. 10.1016/j.jiph.2019.04.011. 31056437 PMC7102841

[18] MERS Situation Update: January 2020. World Health Organization Regional Office for the Eastern Mediterranean. January 2020. https://applications.emro.who.int/docs/EMCSR254E.pdf. Accessed 28 Jan 2021.

[19] Xiao J , Fang M , Chen Q , He B . SARS, MERS and COVID‐19 among healthcare workers: a narrative review. J Infect Public Health. 2020 June;13(6):843‐848. 10.1016/j.jiph.2020.05.019. 32493671 PMC7250777

[20] Lietz J , Westermann C , Nienhaus A , Schablon A . The occupational risk of influenza A (H1N1) infection among healthcare personnel during the 2009 pandemic: a systematic review and meta‐analysis of observational studies. PLoS One. 2016 Aug 31;11:e0162061. 10.1371/journal.pone.0162061. 27579923 PMC5006982

[21] McDonald LC , Simor AE , Su IJ , et al. SARS in healthcare facilities, Toronto and Taiwan. Emerg Infect Dis. 2004 May;10(5):777‐781. 10.3201/eid1005.030791. 15200808 PMC3323242

[22] The IMF's Response to COVID‐19. International Monetary Fund; 6 Oct 2020, updated 28 Oct 2020. https://www.imf.org/en/About/FAQ/imf‐response‐to‐covid‐19. Accessed 28 Jan 2021.

[23] Craven M , Sabow M , Van der Veken L , Wilson M . Not the Last Pandemic: Investing Now to Reimagine Public‐Health Systems. McKinsey & Company; 13 July 2020. https://www.mckinsey.com/industries/public‐and‐social‐sector/our‐insights/not‐the‐last‐pandemic‐investing‐now‐to‐reimagine‐public‐health‐systems. Accessed 28 Jan 2021.

[24] Global Progress Report on WASH in Health Care Facilities: Fundamentals First. World Health Organization; 2020. https://www.who.int/publications/i/item/9789240017542. Accessed 28 Jan 2021.

[25] Allegranzi B , Kilpatrick C , Storr J , et al. Global infection prevention and control priorities 2018‐22: a call for action. Lancet Glob Health. 2017 Dec;5(12):e1178‐e1180. 10.1016/S2214-109X(17)30427-8. 29132606 PMC7129117

[26] Risko N , Werner K , Offorjebe OA , Vecino‐Ortiz AI , Wallis LA , Razzak J . Cost‐effectiveness and return on investment of protecting health workers in low‐ and middle‐income countries during the COVID‐19 pandemic. PLoS One. 2020 Oct 9;15(10):e0240503. 10.1371/journal.pone.0240503. 33035244 PMC7546502

[27] Wilkason C , Lee C , Sauer LM , Nuzzo J , McClelland A . Assessing and reducing risk to healthcare workers in outbreaks. Health Secur. 2020 May/Jun;18(3):205‐211. 10.1089/hs.2019.0131. 32559156

